# Ocean Surface Winds Drive Dynamics of Transoceanic Aerial Movements

**DOI:** 10.1371/journal.pone.0002928

**Published:** 2008-08-13

**Authors:** Ángel M. Felicísimo, Jesús Muñoz, Jacob González-Solis

**Affiliations:** 1 Escuela Politécnica, Universidad de Extremadura, Cáceres, Spain; 2 Real Jardín Botánico (CSIC), Madrid, Spain; 3 Departament de Biologia Animal (Vertebrats), Universitat de Barcelona, Barcelona, Spain; Estación Biológica de Doñana, CSIC, Spain

## Abstract

Global wind patterns influence dispersal and migration processes of aerial organisms, propagules and particles, which ultimately could determine the dynamics of colonizations, invasions or spread of pathogens. However, studying how wind-mediated movements actually happen has been hampered so far by the lack of high resolution global wind data as well as the impossibility to track aerial movements. Using concurrent data on winds and actual pathways of a tracked seabird, here we show that oceanic winds define spatiotemporal pathways and barriers for large-scale aerial movements. We obtained wind data from NASA SeaWinds scatterometer to calculate wind cost (impedance) models reflecting the resistance to the aerial movement near the ocean surface. We also tracked the movements of a model organism, the Cory's shearwater (*Calonectris diomedea*), a pelagic bird known to perform long distance migrations. Cost models revealed that distant areas can be connected through “wind highways” that do not match the shortest great circle routes. Bird routes closely followed the low-cost “wind-highways” linking breeding and wintering areas. In addition, we found that a potential barrier, the near surface westerlies in the Atlantic sector of the Intertropical Convergence Zone (ITCZ), temporally hindered meridional trans-equatorial movements. Once the westerlies vanished, birds crossed the ITCZ to their winter quarters. This study provides a novel approach to investigate wind-mediated movements in oceanic environments and shows that large-scale migration and dispersal processes over the oceans can be largely driven by spatiotemporal wind patterns.

## Introduction

Understanding factors driving large-scale movements of aerial organisms, propagules and particles, is critical to predict the dynamics of migration and dispersal, including colonizations, invasions or spread of pathogens. It is well known that winds are one of the major agents driving movements of any air suspended object, from a particle to an albatross [1]–[4]. This is particularly true in the marine environment, where winds are stronger and more constant than on land, and the apparent lack of barriers facilitate aerial movements. However, much of the evidence is based on global wind patterns and indirect evidences of dispersal or migration movements through the study of extrinsic or intrinsic markers or local measurements [5]–[8]. The combined spatiotemporal component of global movements and winds has not been explored yet in detail due to both, the lack of high-resolution dynamic spatiotemporal wind measurements and the inability to obtain individual tracks of large-scale aerial movements. Nevertheless, recent developments in the study of wind patterns from satellite remote sensing systems as well as the miniaturization of tracking devices provide new opportunities to tackle the interaction processes between winds and aerial movements.

At present, the strength and direction of oceanic winds are available from satellite scatterometers with high spatial and temporal resolution, and global coverage. This information can be used to build anisotropic “cost models” reflecting the resistance to the movement through a wind landscape. Wind cost models have been recently used to demonstrate the importance of winds for long distance dispersal in mosses, liverworts, lichens and pteridophytes [9]. The actual movements of the minuscule propagules responsible for transport in these groups, either spores or fragments, cannot be tracked easily and therefore the importance of winds had to be indirectly inferred from the similarity in species composition among localities [9]. Using a model organism that can be tracked over long periods of time, it would be possible to go a step further and study the interactions between wind-mediated processes and actual individual movements at the same spatial and temporal scales.

Transoceanic movements have one of its most dramatic expressions in bird migration, since millions of birds move between the two hemispheres every year. Miniaturization of data loggers currently provide a wealth of information on spatial (e.g., trajectories, stopovers), and temporal aspects of the migration at individual level [10]. Migratory journeys of pelagic birds are among the longest ever recorded. The use of electronic geolocators has shown that Cory's (*Calonectris diomedea*) and sooty shearwaters (*Puffinus griseus*) travel for tens of thousands of km across the oceans [11], [12]. Such astonishing distances are much longer than the great circle (orthodromic) routes connecting the migration endpoints, and follow global wind patterns, suggesting that birds optimize the use of winds for gliding [1], [13]–[17]. However, most knowledge on this relationship is based on synoptic data or inferred from experimental conditions or local observations, and no attempt to analyze concurrent winds and trajectories has been made so far.

In this study we test the hypothesis that wind strength and direction determine the main spatial and temporal patterns of movements above the oceanic surface. We present an integrative study where wind data collected by an Earth-orbiting satellite is combined with simultaneous information on location of a model organism: the Cory's shearwater, which annual movements, contrarily to most dispersed organisms or particles, can now be tracked by electronic geolocators. To test the spatiotemporal association between optimal pathways defined by wind and actual bird flyways, we used anisotropic cost analysis to build daily wind cost models, which were compared to the tracked routes. Additionally, to test if movements over the oceans are temporally conditioned by calms and strong opposite winds, we checked the role of a putative dispersal barrier: The Atlantic sector of the Intertropical Convergence Zone, or ITCZ [18]. We conclude that large-scale migration and dispersal processes over the oceans are largely driven by spatiotemporal wind patterns, which opens new avenues of research to explore invasion routes or commonly supposed anomalous distributions of organisms that use wind either active or passively for their transport.

## Materials and Methods

### Wind data

The SeaWinds is a specialized microwave radar (scatterometer) aboard the QuikSCAT satellite, developed by NASA JPL to measure near-surface ocean wind speed and direction. Wind stress over the oceans generates ripples and small waves, which roughen the sea surface. These waves modify the magnitude of the backscattered power received at the instrument, which calculates wind parameters from such modification. What makes optimal this instrument to study the flight characteristics of our model organism, is that *Calonectris diomedea* uses the updrafts caused by the wind meeting the rough sea surface to move across wave fronts with the minimum of active flight.

Processed SeaWinds Level 3 data for each day are stored as zipped Hierarchical Data Format (HDF) files, which were downloaded from ftp://podaac.jpl.nasa.gov/pub/ocean_wind/quikscat/L3/data. Each HDF file contains a set of 16 Scientific Data Sets (SDS), including wind power, *u* and *v* components of wind vectors and rain flags. Each HDF SDS is a simple 0.25° rectangular grid of 1,440 pixels from east to west and 720 pixels from south to north in a standard “plate carrée” projection [19], [20]. Parameters of interest for calculation of anisotropic cost (i.e., *u* and *v* components of wind vectors) were extracted from the HDF daily files using an IDL script (available upon request) run in ENVI 4 (www.RSInc.com/ENVI). All subsequent steps of anisotropic cost analyses were run in ArcInfo Workstation (www.esri.com).

### Anisotropic Cost Analysis

**Figure 1:**
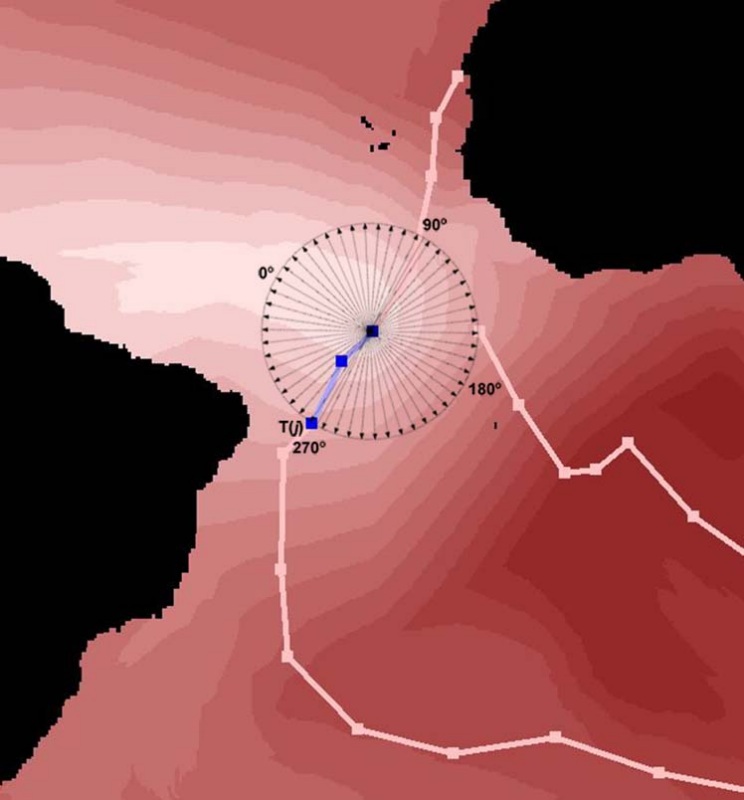
Calculation of the TDTs to test the hypothesis of no association between flight and minimum wind cost directions. The blue segments represent a “two-day trajectory” (TDT). We set 50 points spaced at 7.2° (each defining a vector *T(i))* on the circumference with a centre in the starting locality of the TDT and a radius corresponding to the total distance flight during the TDT. The value of 0° corresponds to the minimum wind cost trajectory. The vector *T(j)* approximates the flight heading, while the rest of vectors represent alternative trajectories not used by the bird. The background color represents the anisotropic wind cost for these TDT days (increasingly higher cost from light to dark saturation).

Anisotropic Cost Analysis estimates the minimum accumulative cost of moving from a source cell to every other cell on a raster model considering direction as a parameter, as opposite to the undirectional isotropic cost (cf. background in Figure 1). In the present study the impedance or friction grid is the wind surface, from which two properties are used to calculate the cost models: speed and azimuth. The final cost is the inverse of the speed multiplied by the *Horizontal Factor* (*HF*), a function that incrementally penalized angular deviations from the wind direction:
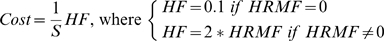
where *S* = wind speed, and *HRMF* = Horizontal Relative Moving Angle, or angle between the moving direction and wind azimuth.

**Figure 2:**
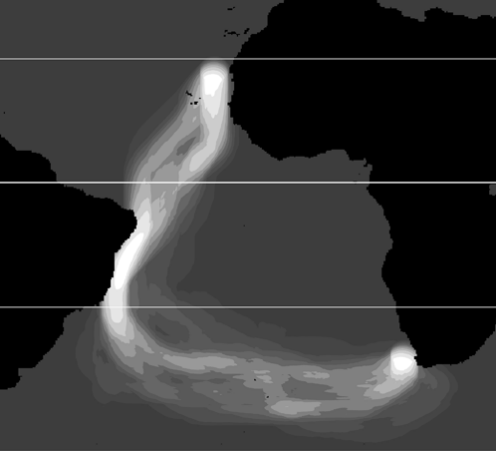
Minimum cost corridors calculated from SeaWinds data corresponding to the migration period of Cory's shearwaters (Nov., 9–30). Increasingly lighter tones represent greater concentration of low-cost corridors (“wind highways”), while very dark tones represent lower concentrations of wind highways and, therefore, unlikely migratory routes between bird's breeding and wintering areas. The thicker line represents the Equator, and the thinner lines, the Tropic of Cancer and Capricorn.

Through anisotropic cost analysis we calculated the minimum cost corridors connecting breeding and wintering areas corresponding to the migration period of Cory's shearwaters (Nov., 9–30) shown in Figure 2.

### Bird tracking

**Figure 3:**
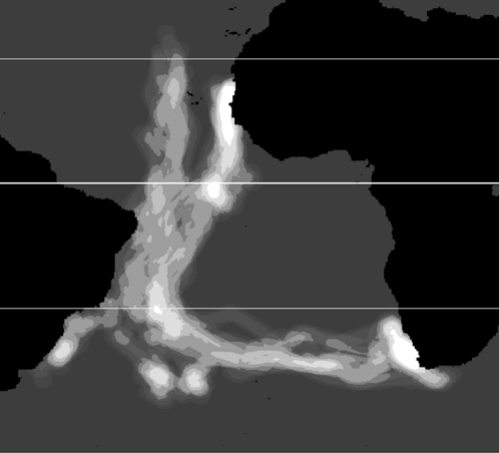
Density kernel of Cory's shearwater flyways. Increasingly lighter tones represent greater concentration of trajectories. The thicker line represents the Equator, and the thinner lines, the Tropic of Cancer and Capricorn.

In June and July 2002, we deployed a total of 50 geolocators at Vila Islet (Azores), Veneguera (Gran Canaria, Canary Islands), and Pantaleu Islet (Balearic Islands, Mediterranean). After approximately 1 year, we recovered complete data from eight, seven, and five geolocators from these three sites, respectively. A preliminary deployment at Chafarinas Island (Mediterranean) in June 2000 yielded data from two geolocators in June 2001. For this study, we choose the 15 transequatorial trips that allow contrasting the hypothesis of connection between wind highways and bird pathways. Some of the recovered geolocators showed incoherent data by a variety of causes, but mainly due to the proximity to the autumn equinox (see below); Figure 3 shows a synthesis of them through Kernel Density Estimation. Deployment of these geolocators on Cory's shearwaters has no detectable short-term effect on the birds [21]. The 10-g geolocators used in this study were developed by the British Antarctic Survey. We deployed them on the leg of each bird, mounted on a DARVIC ring. The geolocator was equipped with an internal clock and measured the light levels every 1 min, recording the maximum reading within each 10-min interval [22]. From this information, two positions per day (one corresponding to midday and the other to midnight) can be inferred with an average accuracy of 186 km±114 km [23]. Positions were calculated using Multitrace-3/16 light (http://www.jensen-software.com/) by inspecting the integrity of the light curve day by day and fitting dawn and dusk times. The elevation angle of the sun was set at –5.5 degrees. To filter unrealistic positions we removed: (1) those obtained from light curves showing interferences at dawn or dusk (mainly due to the bird staying in the burrow), (2) those with a speed index above 35 km hr^−1^, as calculated by the root of the square speed average of the segments formed with the two preceding and the two following positions [24], and (3) those within the equinox periods ranging from March 11 to April 6 and from September 4 to October 13 [11].

### “Two-Day Trajectories” (TDT)

To test whether birds used low wind cost pathways, each of the 15 bird flyways were partitioned into segments representing two-day trajectories (TDT), the minimum time span required to avoid wind no-data values. For each TDT we defined a circumference with center in *O* (starting point of each TDT) and radius *L* (total distance flight during the same TDT), over which we set 50 points spaced at 7.2°, each defining a vector *T(i), i* = 1→50. One of the vectors, *T(j)*, approximates the followed TDT, while the rest represent alternative trajectories not used by the bird (Figure 1). Anisotropic cost analyses were run for the same TDT days, and the cost of moving from *O* to each of the fifty points –*C(i)*, *i* = 1→50– was extracted and ranked, with *C(j)* as the observed cost for that TDT. If birds fly irrespective of wind, we would expect the proportion of *C(i)* above and below the observed *C(j)* to be roughly equal. A binomial test was used to check this assumption.

### Monte Carlo simulations of trajectories

The mathematically calculated minimum wind cost trajectory is that optimizing the cost of the trip considered in its entirety. If the average trajectory followed by the birds were among the 5% more similar trajectories to the minimum wind cost trajectory among all random trajectories having the same endpoints, it would indicate that birds use minimum costs pathways for their transoceanic movements. To test this hypothesis, we used an iterative procedure to generate 10,000 random trajectories over the ocean having the same origin and endpoint shown in Figures 2 and 3; endpoint was defined as the centroid of birds' arrivals. Firstly, we defined the segment joining the fixed origin and endpoints and traced the perpendicular at its midpoint. Secondly, we picked a point on the perpendicular, over the ocean, at a distance defined by a uniform random number generator; this point in fact defined the general shape of that particular random trajectory. Thirdly, this randomly picked point was joined to the fixed points to define two new segments, over which the same procedure was repeated restricting the maximum distance to the length of the newly generated segments, to avoid unrealistic erratic trajectories. Each trajectory was created repeating the above procedure with 15 points besides the fixed endpoints. Lastly, we calculated (1) the mean quadratic distance (MQD) from the average bird trajectory to the minimum wind cost trajectory, (2) the corresponding 10,000 MQDs from each random replicate to the minimum wind cost trajectory, and (3) we ordered the 10,001 MQD values to estimate the *P* value under the null hypothesis of no association between the minimum wind cost path (Figure 2) and the average bird trajectory (Figure 3).

### Crossing wind barriers

Under the hypothesis of wind driving movement, calms or strong opposite winds will act as an invisible gate opening or closing transoceanic paths. In large scale studies, like those using synoptic maps, this phenomenon would pass in most cases undetected, as real wind corridors can be narrowly defined both in space and time. The fine grain of SeaWinds data allows detecting where such “gates” are located, and when they can block or allow dispersion.

**Figure 4:**
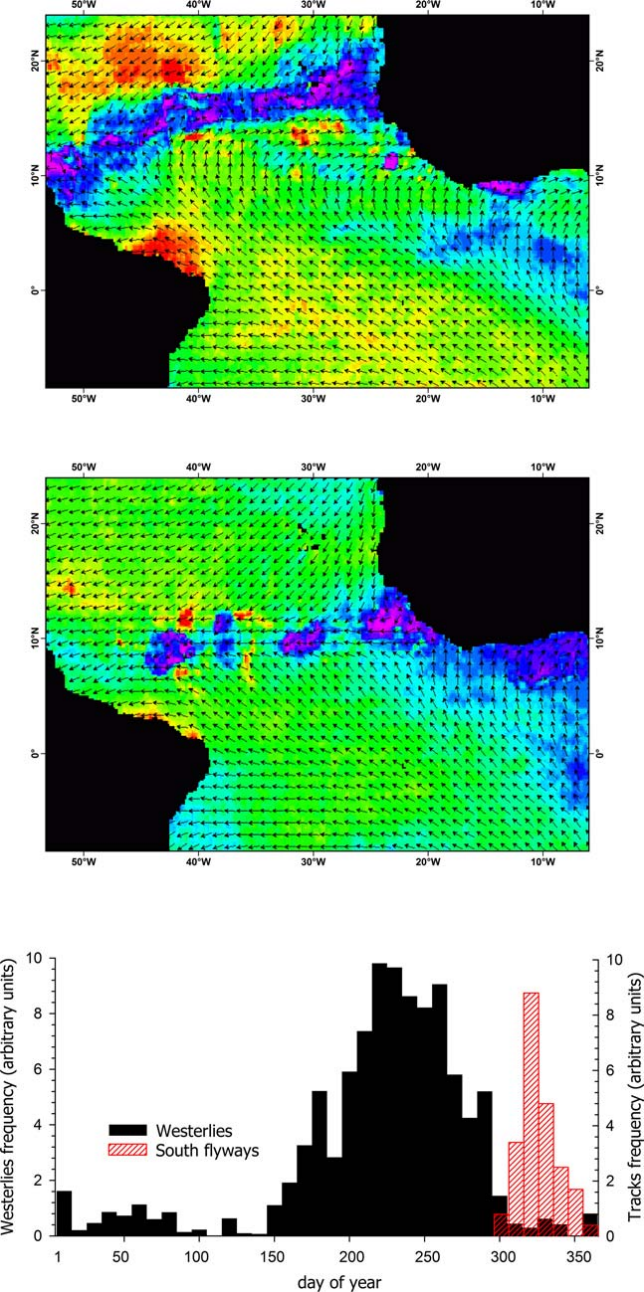
Coupling of Cory's shearwaters migration to the stopping of westerly winds developed during the peak months of the African Monsoon in the Atlantic sector of the Intertropical Convergence Zone (ITCZ). QuikSCAT winds on 7 September 2002 (4A) and 15 November 2002 (4B) showing the migratory “gate” located at 10°–15° N blocked and opened, respectively. Wind speed in the background increases from fuchsia (0 m·s^−1^) to green (6 m·s^−1^) to red (15 m·s^−1^), and arrows show wind direction. 4C, Migration (hatched red bars) starts when the frequency of blocking winds drops (black bars; winds blow towards 75°–115°). In the returning trip (range: February 11–March 10), there are no westerlies blocking the passage to the breeding areas in the Northern Hemisphere (not shown).

The Atlantic sector of the Intertropical Convergence Zone (ITCZ) is a narrow belt of calms girdling Earth over the ocean near the Equator, at the junction of the northeast and southeast trade winds, which moves north and south seasonally [18]. To examine the impact of the seasonal calms and monsoon westerlies associated to the ITCZ as a possible barrier to aerial trans-equatorial movements, we performed two independent analyses: 1) we counted the number of pixels over which birds actually flew that have a wind azimuth in the range 75°–115° (NE to SE), considered to block southbound movements of birds, outside (Figure 4A and 4C, black bars) and during (November 14–16, 2002; Figure 4B and 4C, hatched red bars) the period of bird migration; and 2) we run an ANOVA to compare all pairwise costs of traveling from five points north to five points south of the ITCZ, during (September 6–8, 2002) and after (November 14–16, 2002) the African Monsoon.

## Results

### Wind cost analysis

Anisotropic wind cost analysis applied to the spatiotemporal dynamics of the tracked model showed that the minimum wind cost trajectory is not the great circle line between Canary and Benguela currents, but a SW loop that turns east after crossing the tropic of Capricorn (Figure 2).

### Tracked birds

Most Cory's shearwaters traveled 15,000–35,000 km across much of the Atlantic Ocean in a figure-eight pattern to winter on the Brazil-Falklands, Benguela and Agulhas currents of the Southern Hemisphere before returning to the breeding areas. Birds breeding on Balearic and Canary Islands (*n* = 7) used a narrow corridor centered at 20° W (±3°) and crossed the equator highly synchronized (Nov. 15±6.3 days). Birds breeding on Açores Islands (*n* = 8) used a similarly narrow pathway centered at 27° W (±5°), and crossed the equator ten days later, although equally synchronized (Nov. 24±6 days).

Actual trajectories of tracked birds closely resembled minimum wind cost trajectories. After crossing the equator, all shearwaters headed SW. After crossing the tropic of Capricorn, four birds remained on the Brazil-Falkland current area whereas eleven birds turned east and continued migration until reaching the Benguela current area. Figure 3 shows a synthesis of the 15 southbound trajectories through Kernel Density Estimation.

### TDT analysis

TDT directions are clearly different from random (Rayleigh test: *r* = 0.862, *Z* = 292.908, *P*≪10^−6^), and turned 50°±31° from the minimum cost direction (*V* test, expected mean 0°, *V* = 0.554, *u* = 15.544, *P*≪10^−6^). Moreover, low-cost trajectories (below the 45 cost percentile) represent more than 80% of the observed TDT, while high-cost paths (>55 cost percentile) accounted for less than 8%.

### Monte Carlo simulations

The designed Monte Carlo randomization test demonstrates that visual similarity between minimum wind cost trajectories and the flyways from the tracked birds (Figures 2 and 3) is indeed the expression of a strong relationship (*P*≈0.01), and thus the null hypothesis of no association can be rejected.

### Crossing wind barriers

As usual during the summer African monsoon, prevailing winds in central Atlantic showed a dominant westerly component and formed a low pressure belt from French Guiana to Mauritania-Senegal coast at the Intertropical Convergence Zone (Figures 4A and 4C). In November, monsoon drop and prevailing winds in the central Atlantic turn to a dominant easterly component (Figures 4B and 4C). Birds (hatched red bars) crossed the ITCZ when the frequency of westerly winds (black bars, winds blow towards 75°–115°) drop (Figure 4C). Wind cost models revealed that pathways used by birds during their trans-equatorial migration would be on average ∼60% more costly during the monsoon season than the costs incurred when birds actually crossed the Atlantic sector of the ITCZ (*F*_(1, 48)_ = 35.94, P≪0.0001).

## Discussion

Our model organism, the Cory's shearwater, is a long distance migrant that moves between the breeding and the wintering areas. To achieve this task, actual migration path and timing can be mediated by environmental determinants, in particular by wind speed because this is of the same order of magnitude as the bird airspeed [6], [25]. In consequence, the optimum path may not necessarily be the shortest path. Indeed, Cory's shearwater tracks did not follow the shortest (great circle) trajectory connecting breeding and wintering areas, but flyways were 1.6–3.1 times longer; neither followed geographic or magnetic loxodromes, nor the direct sun compass directions. Trans-equatorial routes described a figure-eight pattern, clock-wise in the northern and counter-clockwise in the southern hemisphere [11]. A roughly similar pattern was first suggested for another pelagic seabird, the short-tailed shearwater *Puffinus tenuirostris*, in the 1950s [26]. Since then, a figure-eight pattern has been repeatedly suggested to describe migrating movements of a number of pelagic birds [26]–[28]. However, the proposed routes contained several errors and uncertainties because they were based on recoveries and sightings, which provides information limited on space and time as well as unrelated to the breeding status, origin or age of the observed birds. Nevertheless, the figure-eight pattern has now been confirmed in two other shearwater species on the basis of individual movements thanks to the very recent developments of tracking systems, such as the global location sensing (GLS) [11], [12].

Whether dispersal and migration movements are influenced by global wind circulation patterns has long been discussed, including the case of the figure-eight pattern of migrating shearwaters [27]. However, to adequately investigate this hypothesis we require both, information from speed and direction of winds and a model which movements can be accurately tracked. The present paper is the first one rigorously testing such hypothesis, because we analyzed both sets of information, concurrent in space and time. Our results demonstrate that local decisions (TDT trajectories) are associated to low cost alternatives and avoid tail winds likely to prevent stalling. Most importantly, global flyways (Figure 3) connecting breeding and wintering areas closely match the minimum cost “wind highways” mathematically calculated from daily wind fields (Figure 2). These results strongly support that global wind patterns are the major responsible for the migration routes followed by shearwaters, and that global flyways can result from decisions taken along the route based on the perception of local wind conditions.

Departure decisions of migrating birds can also be mediated by winds [29], [30]. Our results show that the crossing of the Atlantic sector of the ITCZ was highly organized and occurred when both wind calms and near surface westerlies drop. These results suggest that winds block the southbound migration at 10°–15° N from May to September, during the peak of the African monsoon [18], [31], [32]. Cory's shearwaters fly close to the ocean surface using the wind stress to glide over the waves, which limits their capacity to select other flight altitudes with more favorable winds. Our analysis shows that departure during the African monsoon season would increase migration costs about 60% compared to when Cory's shearwaters actually crossed the ITCZ.

We have demonstrated in a previous paper that wind is the main responsible for the similarity in passively transported organisms among remote areas [9]. In the present paper we show that the distribution of actively flying or gliding animals can also be highly influenced by winds. Although here this is demonstrated for a model organism, we think that this is a general phenomenon that could explain odd distribution patterns. Perhaps the rarity of such patterns is not a reflection of rarity in the wind dispersal phenomenon, which would be in fact common, repeated and predictable [33], but to the conditions that are required later for the establishment and long-term persistence of populations [34].

The strength of our wind cost analyses highlights the importance of gathering simultaneous data from fine scale winds and aerial movements to investigate the complex processes that ultimately derive in a successful long-distance trip. The success of a wind-mediated invasion, migration, colonization or pathogen spread not only depends on the geographic distances between endpoints, but also on wind connectivity. Our results show that winds can promote dispersal between areas that, using large scale synoptic maps, would not be considered as actually connected.
